# A Scoping Review of Physician Advocacy on Twitter

**DOI:** 10.7759/cureus.41632

**Published:** 2023-07-10

**Authors:** Abigail M Cahill, John C Carlson

**Affiliations:** 1 Pediatrics, Tulane University School of Medicine, New Orleans, USA; 2 Pediatrics, Oschner Health System, New Orleans, USA

**Keywords:** physician online presence, residency curriculum, online advocacy, twitter, advocacy

## Abstract

Twitter has been adopted by physicians across most medical specialties; it allows for the wide dissemination of information and calls to action, brings new stakeholders into collations, promotes academic engagement, and fosters collaboration between academia and private practice. In this review of the literature, we briefly outline the state of advocacy in health care and summarize current Twitter-based advocacy efforts in the major specialties of health care, identifying both successful strategies as well as gaps in Twitter advocacy research. Relevant articles were obtained via PubMed and Google Scholar searches using the phrases “Twitter advocacy healthcare,” “[specialty name] Twitter” and “[specialty name] Twitter advocacy.” Several overarching themes were found to be widely utilized in specialty-specific discussions of Twitter advocacy efforts: organizing under a specific hashtag, fostering dialogue between stakeholders, and tweeting using personalized, action-oriented language. Fields such as pediatrics, heme/onc, ENT, and ophthalmology have most thoroughly embraced the desire to learn how to most effectively advocate on Twitter. Other fields such as OBGYN, cardiology, and surgery have less academic focus on online advocacy. Outside of advocacy efforts, the research and academic benefits of Twitter are well described in nearly every specialty. In conclusion, while clinicians are encouraged to advocate online, only broad strategies for online engagement are currently offered. Additional research into the details of how to successfully create an online profile and Twitter presence is needed to ensure all physicians are able to maximize their advocacy efforts, with clarification of the goals and objectives of this engagement also required.

## Introduction and background

With the broad definition of “speaking on another’s behalf” and working within existing systems to promote the voices of others [1], advocacy has long been deeply engrained in the practice of medicine. Its relevance to quality patient care is supported in the literature, as residents take more attuned social histories [2], develop practical advocacy skills, and form meaningful community networks when exposed to advocacy training in their program curricula [3]. The ways in which clinicians advocate have evolved over time and continue to take many forms, including testimony before governing boards, publication of opinion articles, and communication through social media [4].

Social media has been well established as an “important tool for scientific collaboration, education, advocacy, patient engagement, and improved patient care” [5]. In the early days of online advocacy, message forums were used to effectively build early networks of healthcare providers [6]. As discussion boards were replaced by social media platforms, this form of online networking has grown, with social media playing an important role in the shift to collective advocacy in health care [7]. This includes Twitter, for which there have been over 140 reported uses in medicine, ranging from online journal clubs to consults [8]. On Twitter, users may post tweets - messages of 280 characters or less, which appear in a public stream, often called a “feed.” Other users can amplify messages by “retweeting” a post to their own followers and can add comments; the comments can likewise be shared by other users. Messages that provoke massive sharing and comments can reach millions of users, a phenomenon referred to as “going viral.” The use of hashtags on Twitter before a word or phrase makes it searchable, enhancing the visibility of the tweet.

Twitter has been a growing platform for health research, advocacy, and academic efforts [9]. Twitter movements such as #thisisourlane and #getusPPE were initiated by healthcare workers and went viral [10]. #thisisourlane, in which healthcare workers asserted their role in the fight against gun violence, proved that organized social media interventions have the ability to instigate real-world discussions of policy, policy formation and compliance, and policymaker awareness. More broadly, Twitter has been used for civic mobilization efforts in the US and abroad, demonstrating its potential to foster discourse between influential stakeholders, connecting online and offline activities [11]. These connections matter as the behaviors of users in one’s Twitter network have the ability to modulate an individual’s behavior [12]. Twitter may also bring new individuals to the table, adding their voices and perspectives to greater conversations surrounding health care [13]. For example, key players in Twitter pediatric advocacy networks include pediatric healthcare providers, advocates, as well as individuals/parents [14].

In this review, we examine and contrast the Twitter engagement of several of the main medical specialties with a focus on the state of advocacy efforts. We highlight successful organizing strategies employed by different medical communities that may be generalized to other fields. Examples of patient education, medical education, and dissemination of research findings are included in this review for specialties that are not performing more traditional examples of advocacy, as dissemination of information can be a component of advocacy when it is directed to stakeholders unaware of patient needs [15].

## Review

Methods

Articles for this review were obtained via PubMed and Google Scholar, using the following search phrases: “Twitter advocacy healthcare,” “[specialty name] Twitter” and “[specialty name] Twitter advocacy.” Articles that were related to the use of Twitter by the medical profession were reviewed and included depending on whether they had relevance to advocacy. All article types were accepted. For specialties without specific Twitter advocacy-related articles, articles that described network characteristics, types of Twitter engagement, or outcomes of Twitter utilization were included. As Twitter is a worldwide platform, articles were not excluded based on their country of origin but were required to be authored in English.

Use of Twitter by specific groups of physicians 

Pediatrics

Pediatricians have embraced online advocacy efforts by organizing around the hashtag “tweetiatrician” [16]. The hashtag #tweetiatrician has been used to pull pediatricians into dialogue on a range of advocacy-related topics, from legislation to COVID vaccine campaigns. In addition, the neonatology community has built connections using #neoTwitter [17]. An analysis of tweet authors using #neoTwitter found that 66% of tweets were authored by neonatologists and researchers, and another 25% were authored by neonatal organizations. Thus, this hashtag allows for users to easily access and engage in discussions of interest, bringing together the different stakeholders throughout the field. The pediatric urology community has also organized under the hashtag #pedsuro, although this hashtag is not specific to advocacy efforts but rather an overarching hub for academic and professional advancement efforts [18]. For individual accounts, a greater number of tweets per month was associated with a greater number of new followers over the same time period [16], suggesting that frequent sharing of tweets could be used to generate a larger community/base of followers.

Twitter has also been used for educational campaigns to spread information related to sudden infant death syndrome (SIDS) and safe sleep practices [19]. In an analysis of tweets containing keywords related to these topics, Pretorius and colleagues (2018) found that most tweets contained recommendations on safe sleeping and devices, possible causes and protective modulators of SIDS, as well as personal stories. The majority of this information came from news organizations and universities in comparison to public health organizations. 

*Psychiatry* 

In psychiatry, Peters and colleagues identify the potential for psychiatrists to intervene in Twitter’s suicide risk detection mechanisms, as well as substance abuse and mood states, particularly for adolescents and young adults [20]. The authors emphasize that the appropriate behavior standards for healthcare providers on Twitter are less about whether one should share personal or professional content and more simply about whether things that are shared are appropriate for a healthcare professional to post in a public space. Investigations into why Twitter is so heavily used for mental health-related discussions [21], as well as cultural trends [22] and diction [23,24] highlight the capacity for psychiatrists to use Twitter for collaborating with patients and other mental healthcare providers.

Neurology

Mishra and colleagues provide one of the only peer-reviewed papers on the use of Twitter in neurology [25]. Neurology journals, individual healthcare providers, researchers, and even pharmaceutical companies are active on Twitter. Many such neurology-related tweets are organized around #neurotwitter, but other more specific hashtags, such as #ALSResearch and #PMSF, are used to increase disease and research visibility for rare diseases, as well as supplement study recruitment [25]. Future work is required to ascertain the effectiveness and varying strategies of advocacy in the neurology community, as there could be a great deal of overlap between neurology-related advocacy and various disability advocacies.

Radiation oncology

In radiation oncology, Twitter is primarily used for the dissemination of information between academicians. #radonc is used by professional societies with a hub-and-spoke broadcast network wherein posts from a few accounts are shared widely by many other users [26]. In an analysis of the engagement of different users within this hashtag, accounts that were deemed the most influential were more likely to be male attending physicians in a US-based academic practice. The dominance of professional societies extends to other radiological subspecialties, with interventional radiology societies having increased their followings by 55% in the last two years, perhaps as a result of posting more tweets per day than diagnostic radiology specialties [27].

Researchers have also leveraged Twitter to promote recent publications, as a correlative analysis between the number of Tweets and the number of academic citations revealed a significant positive correlation between the two [28]. This suggests that radiation oncologists are effectively using Twitter to create engaging academic communities that have a real-world impact on research. 

Dermatology

Dermatologic tweets are dominated by personal and educational content. Analysis of a large sample of acne-related tweets revealed that personal tweets were twice as prevalent as tweets pertaining to celebrities or educational dissemination [29]. The most common questions posed in this community were variations of “What is acne” and “Why does acne exist,” highlighting the patient-driven desire for engagement on Twitter by knowledgeable experts. Further evidence of the popularity of using Twitter to discuss personal dermatologic concerns can be found in a review of high-precision tweets collected using dermatological keywords. 65-75% of the collected tweets were related to personal experiences with various skin conditions, most of which were related to eczema, as it is one of the most prevalent dermatologic conditions [30]. 

While many dermatologists have amassed large followings on social media, prominent dermatologic influencers are more likely to be females living in metropolitan areas, focusing on cosmetic dermatology in private practice [31]. Similar to the Sarker dataset previously discussed, Sierro and colleagues found that the content shared by the 10 most prominent dermatologic influencers was primarily composed of personal posts (53%), followed by educational content (43%), with advertisements comprising the smallest category. 

Concerning academic engagement, a 2014 review found that most dermatological journals, professional organizations, and patient-centered organizations were using social networking sites, including Twitter, Facebook, and LinkedIn [32]. With the increasing popularity of such social networks, rates have likely increased.

Otolaryngology

The landscape of Twitter in the otolaryngology field has evolved significantly over the past five years. With limitations on in-person opportunities for the residency application process, there has been a growing academic presence on Twitter [33]. Of the 55 Twitter accounts operated by ENT departments and residency accounts, 36.4% were opened in the 2020 calendar year alone. Higher-ranked programs have been found to be more active on social media than those in lower quartiles, have more followers, and opened their accounts earlier than other programs [34]. The content of such residency-based accounts is predominately centered around team member spotlights, education/advocacy topics, research, and lifestyle/hobbies [35]. As in other specialties, engagement on Twitter is beneficial for otolaryngology journals, as most ENT articles are shared on Twitter [36]. Journals with active Twitter accounts had greater real-world academic influence [37]. Additionally, if authors tweeted their own article, there was an 8.4-citation increase in citations [36], another example of the real-life correlates of online activity for research.

Advocacy-specific efforts in the ENT field have been centered around the hearing loss community, with the majority of Twitter accounts belonging to for-profit or commercial organizations [38]. In a content analysis of tweets published by residency programs, the majority of information shared was targeted toward other healthcare professionals and researchers, with 24.31% of tweets targeted toward the public and/or patients [39]. While these tweets may have contained information appropriate for the public, the majority of interactions with these tweets were still by healthcare professionals, indicating an opportunity for academic programs to improve their advocacy engagement with stakeholders. 

Ophthalmology

Twitter has been recognized as a valuable tool by the field for over a decade, as an early call to Twitter published by Micieli and Micieli in 2012 highlights the many benefits, for journals, professional organizations, and for advocacy [40]. They explicitly highlight the opportunities for advocacy, noting that many politicians, leaders, and news sources are engaged on Twitter. This provides ophthalmologists (and healthcare providers in general) an opportunity to ethically engage with such individuals and organizations, as all interactions would be open to the public. Not only does this enhance transparency and accountability for organizations and news outlets, but it also permits others to see and join in on advocacy efforts. Twitter also permits patients to share their own experiences and opinions regarding their care. While the characterization of patient posts has been limited to Instagram so far, similar methods could be applied to investigate patient sharing on Twitter [41].

As of 2015, relatively few ophthalmology journals (18.7%) and members of the International Council of Ophthalmology (12.8%) were present on Twitter; however, patient advocacy groups were more popular than journals or other academic organizations [42]. Twitter usage, particularly among professionals, has increased over the years with more organizations, individuals, and students engaging as a result of the COVID-19 pandemic [43]. Like in other fields, the powerful benefits of academic networking are present in ophthalmology, as articles tweeted at least once had a 1.7-fold increase in their Google Scholar scores [44]. This benefit can especially be exploited by open-access articles, as Twitter engagement was 2.1x higher in open-access articles compared with articles that were not open-access. Especially relevant in ophthalmology, the ability to share images is a benefit in developing the academic utility of Twitter [45]. However, caution must be exercised as the dissemination of information regarding eye health has the potential to be tied to sponsored content more so than other specialties. 

OBGYN

Interest in the use of social media in the field of OBGYN has recently increased in the literature over the last several years and was a popular topic at the 2020 American College of Obstetricians and Gynecologists (ACOG) meeting. At that conference, Noureddine and Chappelle quantified engagement on Twitter. They found that in September 2019, the majority of tweets published using the keywords “obgyn” and “obstetrics and gynecology” were authored by non-healthcare professionals, of which 85.6% were opinion-based and only 9.2% relayed educational content [46]. In contrast, 14.9% of the tweets authored by healthcare professionals were opinion-based and 38.3% were educational tweets. As topics related to OBGYN practice have become increasingly politicized and legislated, there are many opportunities for OBGYNs to use their Twitter presence for advocacy. As highlighted by Good and Tanouye, the responsibility falls on the healthcare community to drive accurate health information sharing and engagement on social media platforms, including Twitter, since women’s health is particularly susceptible to misinformation campaigns [47]. However, as of 2019, only 38.3% of editorial board members in the six journals with the highest impact factor were active Twitter users [48]. While ACOG and the American Board of Obstetrics and Gynecologists have social media accounts, there remains great potential for improvement, for example via organizing hashtags such as #InternationalWomensDay, #SheforShe, and #HeforShe [47].

The academic benefits of Twitter have been relatively less recognized in the field of OBGYN compared to other fields. While the medical education tool “ObGyn Delivered” has been created and tested on various platforms including Twitter, this tool is targeted at undergraduate medical education [49]. As of 2021, only 15% of OBGYN residency programs had a Twitter account [50], highlighting the potential for greater overall Twitter engagement in all aspects of the field. 

Surgery

In a 2021 analysis of the top general surgery influencers on Twitter, 50% of influencers were attending general surgeons, 3% were general surgery fellows, and 31% were general surgery residents [51]. The vast majority (93%) of attending surgeons had completed a fellowship, most frequently in surgical oncology, laparoscopic surgery, critical care/trauma, and colorectal surgery. Eleven percent of influencers were physicians in non-surgical fields such as emergency medicine and anesthesiology, and 3% of influencers were not physicians but rather researchers or physician assistants. However, there is no qualification for the ways such influencers leverage their platforms. 

While seemingly a specialty that would have less opportunity for remote supplementation of care, Leow and colleagues advocate for the use of Twitter as a means for providing diagnostic assistance to surgeons and patients in low-resource settings [52]. In this capacity, Twitter can be used to provide more equitable care and build community among patients and physicians around the world. It can also be leveraged to build community between physicians, advocate for underrepresented surgeons and trainees, and avoid burnout [53].

Beyond advocacy, surgery has heavily embraced the use of Twitter for academic purposes. Trauma and orthopedic surgery journals with Twitter accounts have higher impact factors; Twitter follower count is positively associated with impact factors [54]. Twenty-five percent of surgery departments have dedicated Twitter accounts, with university-based programs comprising the majority of existing accounts [55]. A “gamified microblogging project” based on Twitter has even been created that significantly improved the American Board of Surgery In-Service Training Exam (ABSITE) percentile ranks of participants over a year [56]. For medical students, an account that shared surgical facts on Twitter had an overwhelmingly positive reception from students when asked about their educational experience [57].

Cardiology

The field of cardiology has embraced the academic utility of Twitter. This includes building awareness and interest in conferences [58], providing support and community among women in the field [59], and advocating for academic leaders to build a presence on Twitter [60]. Respected clinician-researchers [61], as well as the former president of the American College of Cardiology, Dr. Mary Walsh, recognized the potential several years ago [62]. Since then, others have published guides on how medical professionals [63] and researchers [64] can most effectively engage on Twitter. The practical recommendations provided by such articles may be one of the reasons Twitter has become so widely utilized in this field. 

While there are no publications on traditional advocacy efforts on Twitter in the field of cardiology, there is growing recognition that Twitter can be utilized to survey perceptions, knowledge, and topics of engagement among users outside of the medical field [65,66]. Such methodologies could be leveraged to provide uniquely tailored advocacy efforts and education, working with communities most open to them.

Heme/Onc

Hematology and oncology fields have been primarily organized using hashtags [67]. Disease-specific cancer-related hashtags were created intentionally to promote collaboration between healthcare providers, patients, caregivers, and researchers across different types of cancer [68]. There has been a remarkable success in engagement within these communities, as analyses show high levels of engagement within multiple myeloma (#MMSM), leukemia (#leusm), and myeloproliferative neoplasms (#MPNSM) communities [69]. As an example, the myeloproliferative neoplasm hashtag was rolled out in 2014 and allowed patients, researchers organizations, and caregivers to unite and discuss everything from symptom burdens to clinical trial enrollment to novel therapeutic interventions [70]. Taking a patient-centered approach, other hashtags are tailored to certain patient demographics, such as young adults with cancer, to connect patients and facilitate peer support [71]. However, Twitter communities are not limited to use by younger individuals. Geriatric hematology and oncology hashtags organize on the other end of the age spectrum, although community engagement is driven by healthcare professionals networking and sharing new academic advances [72]. 

While less proliferative than the field of oncology, hematology has a growing presence on Twitter [73]. With less of a focus on patient engagement, hematology-related content is dominated by academic societies and individual providers/researchers [74]. While some providers share personal anecdotes or opinions on “hot topics,” the majority of content is focused on hematology. However, the extent to which personal vs. professional content is shared has not been quantified. Disease-specific hashtags have been used by the hemophilia community, driven by advocacy and support groups as well as individuals [75]. Engagement in this topic is highest among individuals with bleeding disorders and non-physician providers. 

Pathology and other specialties

Additional specialties have active roles on Twitter, using the platform to disseminate information. For example, pathology has paid special attention to the teaching capabilities of Twitter. Similar to the teaching taking place between pathologists using a multi-headed microscope in person, Twitter can be used to increase equity in education, as images may be shared and discussed in threads [76]. Additionally, the live tweet feature provides alternatives to in-person attendance at lectures and conferences [77]. The use and network characteristics of pathology Twitter and other specialties have not been thoroughly examined and host a rich potential to explore trends in image-sharing and academic collaboration. Publications available for other specialties focus on the dissemination of information alone, rather than coalition building and system change, which is therefore beyond the scope of this review. 

Interdisciplinary efforts

While most physician advocacy on Twitter has been studied in specialty-specific networks, there are examples of specific causes for which multidisciplinary teams of physicians on Twitter have worked together, namely #thisisourlane and #getusPPE. As previously mentioned, #thisisourlane sought to unite physicians, regardless of specialty, to assert the collective role of healthcare providers working to prevent gun violence. While #getusPPE was created by emergency medicine physician Dr. Esther Choo, it rapidly spread to include all healthcare workers who were treating patients during the COVID-19 pandemic. Tweets created using these healthcare-led hashtags were analyzed by Ojo and colleagues (2021), comparing the language used in tweets containing these healthcare-led hashtags to a control hashtag [10]. The tweets that contained healthcare-led hashtags had more positivity and action-oriented language than the respective comparison hashtags on the same subject. #thisisourlane was more likely to discuss the advocacy and research of healthcare professionals, as well as mention public health and the greater community. However, such tweets were less likely to mention political groups. #getusPPE tweets contained more action-oriented language, while tweets containing either #getusPPE or #thisisourlane were associated with health, positive emotions, and group identity/affiliation. These collections of tweets demonstrate the usefulness of action-oriented language in online advocacy and the power of using community ties to bridge the online and real worlds. Such successful strategies employed by physicians in these interdisciplinary efforts and across all specialties lend opportunities for improvement and further collaboration (Table 1). 

**Table 1:** Summary of strategies employed by Twitter users from all specialties and organizations.

Useful strategies for online advocacy
1) Use action-oriented language
2) Author original tweets in addition to retweeting
3) Leverage group identity/affiliation
4) Build a larger following/community base by tweeting more
5) Create and use hashtags
6) Do not be afraid to share personal content
7) Share links to other articles/websites
8) Connect with nonprofits/other organizations

Academic physicians

Twitter is a valuable place for open-access medical education that can be easily updated and shared with set groups [78]. Additionally, as residency interviews remain online, pediatric program directors are encouraged to have an active Twitter presence [78], a movement echoed by many specialty fields [79]. Medical education and increasing visibility of training programs are not themselves advocacy, but they do enhance the networks available for advocacy work. Twitter promotes collaboration and greater crossover between academia and private practices [78], which could make advocacy efforts more inclusive and well rounded in terms of the providers represented.

Applications for residency curricula

Twitter has become an important communication tool for physician advocates. In 1996, the Pediatric Residency Review Committee of the Accreditation Council for Graduate Medical Education (ACGME) added a requirement for pediatric residents to have structured educational experiences to “prepare residents for the role of advocate[s] for the health of children within the community” [80]. While only pediatric trainees receive protected time for advocacy training, the ACGME common program requirements applicable to all specialties include the core requirement of “advocating for quality patient care and optimal patient care systems” [81]. A structured curriculum is needed to clarify the role of information dissemination and coalition building in advocacy versus outcomes in patient education, medical education, and research.

Twitter advocacy by other organizations

There exists great potential for effective advocacy through partnerships with a variety of individuals and organizations on Twitter. In 2012, Lovejoy and Saxton utilized an information-community-action classification scheme for tweets from the 100 largest nonprofit organizations with Twitter accounts. The majority of such tweets contained information (58.6%), while 13.2% of tweets gave recognition or thanks, and 8.2% of tweets were replies to other messages [82]. The authors thus concluded that three main functions were served by such activity on Twitter: spreading information, fostering dialogue and community, and mobilizing supporters. Similarly, other health organizations (as defined and listed by the U.S. Department of Health and Human Services) primarily provided informational material on Twitter (77%), with 12% of tweets offering emotional support and 8% providing instrumental support via tangible aid [83]. In doing this, social media has been particularly useful in providing generalized support to populations instead of individualized assistance, increasing the efficiency of an organization’s advocacy efforts [20]. However, this is in turn balanced by the downside of information primarily flowing in a unidirectional manner, with less personalized information delivery.

The successful advocacy of environmental scientists in the Flint Water Study provides an interesting and relevant case study for advocacy in health care. These scientists used a wide variety of advocacy techniques throughout their tweets, relying most heavily on public education (34.8% of tweets), expert testimony (21.6% of tweets), and dissemination of science/research (13%) [11]. When looking at the types of tweets they employed, 68% were original posts, 30% were retweets, and less than 2% were replies. For those original posts, a majority (86%) contained links to the scientists’ websites or photos. Notably, only 20% of tweets contained hashtags, unlike many of the current healthcare organizing efforts that focus on hashtag use. This strategy, bringing together individuals from different backgrounds and perspectives in order to share information and build coalitions around calls to action, offers a valuable beginning playbook for how scientists can capture the attention of the public and promote real-world action.

Twitter etiquette 

As more healthcare professionals become involved in online networking activities, questions have emerged surrounding the “proper” behavior of providers on platforms such as Twitter. Many policies are currently rooted in exercising “common sense” while online; however, as Pershad and colleagues assert, more specific guidelines are necessary [84]. Maintaining professionalism, being authentic and having fun, asking for help, and grabbing attention while focusing on and engaging other users have been offered as four principled guidelines for healthcare professionalism on social media [8], which offer a solid framework for what to keep in mind when building a social media following. While it makes sense that the most proficient and experienced doctors online may have the greatest number of followers [84], there is often little guidance on how to gain that experience and following.

There are five general categories of communicative strategies that have been broadly used by Twitter activists from various disciplines, which include information, symbolic activities, organizing, litigious activities, and civil disobedience activities [11]. When constructing tweets, the use of embedded links or media is a common technique employed by communication scientists, as is interaction with users in the network, as many tweets are also constructed as replies to other tweets [85]. Additionally, the use of personal details or perspectives is also commonly utilized, specifically in broadly healthcare-related tweets [86]. 

Further insight into how tweets can be most effectively composed can be gained from a recent experimental study on the different strategies for the dissemination of COVID guidelines. Tweets that had the highest perceived message effectiveness, as well as perceived attitude effectiveness and the highest likelihood of sharing were tweets authored by physicians that contained a personal message [87]. This was in comparison to tweets authored by federal officials and/or that contained an impersonal message. Most notably, however, the presence of personal (as opposed to impersonal) content had a stronger effect than the physician (as opposed to federal official) messenger for perceived message effectiveness, indicating that physicians employing their stories/ideas may be the most effective in conveying guidelines or recommendations.

As the primary downside of Twitter is the ability for anyone, regardless of their credentials, to share health-related information with varying degrees of truthfulness, the presence of licensed healthcare providers to provide accurate, evidence-based information is of increasing importance [14]. Because Twitter is a relatively unregulated platform, the accuracy of the information shared must always be considered before sharing [88]. Additionally, care must be taken, particularly when sharing clinical cases, to avoid violations of patient confidentiality, as even though a tweet may be deleted, there are few ways to control its dissemination on the internet.

## Conclusions

There are commonalities seen in using Twitter for advocacy across multiple specialties. The use of hashtags used to build communities of physicians within specialties (as done in hematology/oncology and pediatrics) and between specialties (ex: #getusPPE; #thisisourlane) warrants prospective study. Likewise, strategies to expand those coalitions to include non-physician stakeholders should be incorporated into training. There are differences in outcomes demonstrated for different groups of physicians; exploring similarities and differences across specialties may further direct the curriculum to enhance the effectiveness of physician advocacy on Twitter.
